# Phylogenetic Analyses of Bostrichiformia and Characterization of the Mitogenome of *Gibbium aequinoctiale* (Bostrichiformia Ptinidae)

**DOI:** 10.3390/genes16050509

**Published:** 2025-04-28

**Authors:** Hongli Zhang, Zhiping Han, Rui Zhang, Yongfang Zhang, Juan Wu, Zhichao Wang

**Affiliations:** 1 School of Agriculture and Life Science, Shanxi Datong University, Datong 037009, China; 13620629501@163.com (Z.H.); ruiz0106@163.com (R.Z.); zyf_208@163.com (Y.Z.); juanwu0510@163.com (J.W.); lyl_kgylu@163.com (Z.W.); 2 Datong Key Laboratory of Organic Dry Farming for Special Crops, Shanxi Datong University, Datong 037009, China

**Keywords:** Bostrichiformia, *Gibbium aequinoctiale*, mitogenome, phylogenetic analyses, Ptinidae

## Abstract

Background: Ptinidae, within the infraorder Bostrichiformia, are a cosmopolitan, ecologically diverse but poorly known group. The phylogeny within Bostrichiformia and the monophyly of Ptinidae and its phylogenetic placement in Bostrichiformia remain contentious. Methods: In this research, we determined the entire mitochondrial genome (mitogenome) of *Gibbium aequinoctiale*, the first representative mitogenome of the subfamily Ptininae, and reconstructed the phylogenetic relationships for Bostrichiformia based on four mitochondrial datasets using maximum likelihood (ML) and Bayesian inference (BI) methods. Results: The mitogenome of *G. aequinoctiale* is a circular molecule spanning 17,020 bp and harbors 37 mitochondrial genes and a presumed control region (CR). The mitogenome exhibited a marked preference for the utilization of A and T bases, which was also observed in three kinds of genes and CR. AAT was inferred as the putative candidate initiation codon for cytochrome oxidase subunits 1 (*COI*). The control region contains three tandem repeats (TDRs) and one poly-thymine stretch (Poly-T) in both coding strands. The phylogenetic results appeared to support the monophyly of four families, Nosodendridae, Derodontidae, Dermestidae, and Bostrichidae, and the basal position of the latter two families within Bostrichiformia. However, the family Ptinidae was not verified as monophyly because of one species diverging from the main lineage. Three families, Dermestidae, Bostrichidae, and Ptinidae, clustered as the major clade in Bostrichiformia, among which Bostrichidae and Ptinidae grouped together as sister groups. Conclusions: The present study provides valuable mitochondrial information for Ptinidae and provides novel perspectives on the inner phylogeny within the infraorder Bostrichiformia.

## 1. Introduction

The smooth spider beetle *G. aequinoctiale* is a group mainly distributed in warm climes and principally feeds on a wide variety of dead organic materials in the family Ptinidae, which encompasses roughly 230 genera and over 2200 species recognized globally [1,2]. Ptinidae are found throughout the world, and taxa within Ptinidae are currently categorized into 11 subfamilies, with their habits exhibiting an exceptionally high degree of diversity for the relatively small scale of the group [3].

Most insect mitogenomes sequenced exhibit a double-stranded, compact circular DNA structure, with sizes spanning approximately 15 to 18 kb. Mitogenomes comprise the standard set of 37 mitochondrial genes, encompassing 13 protein-coding genes (PCGs), 22 transfer RNA genes (tRNAs), and two ribosomal RNA genes (rRNAs). Additionally, they contain a noncoding control region, which is thought to play an important role in initiating both transcription and replication processes [4]. Mitogenomes are generally inherited through the maternal lineage and are characterized by a conserved structure, a comparatively rapid evolutionary rate, and minimal genetic recombination events [5]. In particular, mitogenomes have frequently been employed for phylogenetic and population genetic analyses due to the ease of their amplification and the rich information they offer across various evolutionary levels [6,7]. Numerous mitogenomes have been sequenced and have been widely employed to elucidate deep-level phylogeny within Coleoptera, especially for the suborder Polyphaga [8,9,10,11,12,13,14,15,16,17,18]. The infraorder Bostrichiformia in Polyphaga originally included two superfamilies, Derodontoidea (Derodontidae) and Bostrichoidea (Anobiidae, Bostrichidae, Dermestidae, Endecatomidae, Nosodendridae, and Jacobsoniidae). Later, Bostrichiformia was divided into two distinct series: Derodontiformia, including three families (Jacobsoniidae, Nosodendridae, and Derodontidae), and Bostrichiformia, including four families (Endecatomidae, Dermestidae, Bostrichidae, and Ptinidae) [19,20]. Ptinidae was previously divided into two families [20], later, the two families were consolidated into a single, unified family, Anobiidae. The family name Ptinidae was preferred for use based on priority and has been accepted by most beetle workers [20]. Until now, the inner phylogeny for Bostrichiformia and the phylogenetic relationship among Ptinidae have remained controversial and have not been thoroughly addressed, especially the molecular phylogenetic implications. Mitogenomes have been confirmed to be exceptionally potent markers and have been widely used to conduct in-depth investigations into the phylogenetic relationships of Coleoptera across diverse hierarchical levels [8,9,10,11,12,13,14,15,16,17,18]. So far, 40 almost complete mitogenomes have been released to the public in the NCBI database (https://www.ncbi.nlm.nih.gov/, accessed on 1 January 2025), with less focus being on determining the inner phylogeny for Bostrichiformia.

In this research, the entire mitogenome of *G. aequinoctiale* (Coleoptera: Polyphaga: Bostrichoidea: Ptinidae) is reported. We focus on preliminary analyses of the characteristics of this mitogenome sequence, including the architecture, nucleotide content, codon preference for PCGs within the mitogenome, and the secondary structure of tRNAs, as well as crucial elements in the CR. Furthermore, four mitogenome datasets were used to obtain insight into the phylogeny among families within Bostrichiformia.

## 2. Materials and Methods

### 2.1. Specimen Sampling and DNA Isolation

An adult *G. aequinoctiale* sample was collected in Shaanxi, China. It was preserved in 99.5% ethanol solution and placed in storage at −20 °C to ensure the integrity of DNA. The entire DNA was isolated from the muscle of the sample, and this extraction process was conducted by employing a TIANamp Micro DNA Kit (Tiangen Biotech (BEIJING) Co., Ltd., Beijing, China) according to the detailed instructions provided by the manufacturer.

### 2.2. Mitogenome Amplification and Sequencing

The mitogenome of *G. aequinoctiale* was amplified as overlapping polymerase chain reaction (PCR) fragments, employing a set of universal primer pairs [21] and two species-specific primers that were designed based on the newly obtained sequence fragments (Table S1). These short fragments were amplified, employing 2 × Taq PCR StarMix with loading dye (GenStar Biosolutions Co., Ltd., Beijing, China), and the reaction was conducted under the following conditions. Initially, the DNA was denatured for 5 min at 95 °C, followed which 35–40 cycles were performed, where each cycle involved a 20 s denaturation at 93 °C, a 30 s annealing at 48–57 °C, and a 1–3 min extension at 72 °C, with a subsequent final 10 min extension at 72 °C. The PCR-generated products underwent purification using the DNA Gel Purification Kit (BioTeke Biotechnology Co., Ltd., Beijing, China), and all amplified products were then directly sequenced from both strands with a primer-walking strategy (Sangon Biotech (Shanghai) Co., Ltd., Shanghai, China).

### 2.3. Mitogenome Assembly, Annotation, and Analyses

Raw sequence files underwent proofreading and were assembled into contigs by employing the Staden package v1.7.0 [22]. Subsequently, the assembled sequence fragments (contigs) were carefully examined for ambiguous base assignments, and only the regions with clear base assignments were employed for annotation purposes. Most tRNAs were identified by employing tRNAscan-SE v1.21 [23]. Subsequently, the remaining tRNAs, along with two rRNAs and 13 PCGs, were recognized by conducting sequence alignment with genes originating from other Polyphaga species [8]. Sequence data have been submitted to and archived in the NCBI database (KY549398). The circular diagram of the mitogenome was visualized by employing CGView (https://stothardresearch.ca/cgview/xml_overview.html, accessed on 22 March 2025) [24].

Nucleotide composition, along with amino acid and relative synonymous codon usages (RSCUs), was ascertained through the application of MEGA v5.1 [25]. We calculated the nucleotide composition and its bias using these formulas: AT% = (A% + T%)/(A% + T% + C% + G%); AT-skew = (A% − T%)/(A% + T%); GC-skew = (G% − C%)/(G% + C%) [26]. The start and stop codons for each PCG were identified through alignment with homologous mitochondrial genes of closely related species. We predicted the secondary structures of tRNAs by tRNAscan-SE v1.21 using the mitochondrial genetic code of invertebrates and default parameters [23]. The search for TDRs within the CR was conducted by utilizing Tandem Repeats Finder v4.0 [27]. The possible hairpin configurations within the repeat unit and the adjacent Poly-T flanking region were forecasted using Mfold v3.1.2 [28].

### 2.4. Phylogeny Reconstruction

The phylogenetic framework within the infraorder Bostrichiformia was reconstructed based on 40 mitogenome sequences, encompassing one species from Derodontidae, two species from Nosodendridae, five species from Bostrichidae, 15 species from Ptinidae, and 17 species from Dermestidae. Additionally, two species from Adephaga (*Rhyzodiastes puetzi* and *Dineutus mellyi*) were incorporated as outgroups for the purpose of rooting the phylogenetic tree (Table 1). In the present investigation, 13 PCGs and two rRNAs were derived from the mitogenome. Phylogenetic studies were performed based on four distinct datasets: (1) PCG (13 PCGs, comprising 11,295 nucleotides), (2) mtDNA (13 PCGs and two rRNAs, comprising 13,652 nucleotides), (3) PCG12 (13 PCGs, excluding the third codon position, comprising 7530 nucleotides), (4) mtDNA12 (PCG12 and two rRNAs, comprising 9887 nucleotides).

Prior to the reconstruction of the phylogenetic tree, sequence alignments were carried out. Each of the 13 PCGs was aligned based on amino acid sequence alignment by MEGA v5.0 [25], and each rRNA gene was aligned with Clustal X v1.83 [29]. Subsequently, the alignments of genes were joined into a single, integrated matrix using Bioedit v7.0 [30]. Segments with gaps and ambiguous positions were removed from the alignments by employing Gblocks v0.91, applying its default parameter settings [31].

A partitioning strategy was implemented across the four datasets, with separate partitions assigned to the codon positions (1st, 2nd, and 3rd) of the PCGs and RNAs. IQ-TREE v2.4.0 was utilized to assess the most suitable evolutionary model for each of the four datasets [32]. Based on the Akaike information criterion (AIC), the optimal model was identified as GTR + F + I + R for the 1st codon position of the PCGs and rRNAs, while TVM + F + I + R was determined as the most suitable model for the 2nd and 3rd codon positions of the PCGs. According to the Bayesian information criterion (BIC), the most appropriate model was determined to be GTR + F + I + G for all three codon positions of the PCGs and rRNAs. The supermatrix (alignment datasets) are presented as Data.zip in the Supplementary Materials.

The reconstruction of phylogenetic relationships within Bostrichiformia was performed through BI and ML methodologies. The BI analyses were performed using MrBayes v3.1, employing four independent Markov chains that were executed over a cumulative duration of one million generations. Samples were collected at intervals of 1000 generations, excluding the first 25% of the sampled trees as burn-in [33]. The ML analyses were carried out by utilizing IQ-TREE v2.4.0, and each node’s support was evaluated by means of 2000 bootstrap replicates [32]. The phylogenetic trees were then visualized using TreeGraph v2.0 [34]. The original phylogenetic trees are presented as Data. Zip in the Supplementary Materials.

**Table 1:** Mitogenome information used in inferring the phylogeny of Bostrichiformia.

Family	Subfamily	Species	ACC.NO	Integrity	Lentgh (bp)	References
Bostrichidae	Dinoderinae	*Rhyzopertha dominica*	NC_042820	Circular	15,859	[35]
Bostrichidae	Dinoderinae	*Dinoderus minutus*	KX087284	Circular	15,230	unpublished
Bostrichidae	Bostrichinae	*Apatides fortis*	NC_013582	Circular	16,171	[8]
Bostrichidae	Bostrichinae	*Sinoxylon* sp. *SIN01*	JX412742	Linear	14,275	unpublished
Bostrichidae		*Bostrichoidea* sp. *KM-2015*	KX035220	Circular	15,462	unpublished
Dermestidae	Dermestinae	*Dermestes dimidiatus*	NC_067497	Circular	16,073	[36]
Dermestidae	Dermestinae	*Dermestes vorax*	NC_086760	Circular	15,775	unpublished
Dermestidae	Dermestinae	*Dermestes ater*	NC_053877	Circular	15,812	[37]
Dermestidae	Dermestinae	*Dermestes lardarius*	NC_053876	Circular	15,772	[37]
Dermestidae	Dermestinae	*Dermestes maculatus*	NC_037200	Circular	16,390	[38]
Dermestidae	Dermestinae	*Dermestes coarctatus*	NC_044851	Circular	15,873	[39]
Dermestidae	Dermestinae	*Dermestes frischii*	NC_044850	Circular	15,873	[39]
Dermestidae	Dermestinae	*Dermestes tessellatocollis*	NC_044849	Circular	16,218	[39]
Dermestidae	Megatominae	*Thaumaglossa rufocapillata*	NC_084110	Circular	16,026	[40]
Dermestidae	Megatominae	*Anthrenus verbasci*	KX087239	Linear	15,818	unpublished
Dermestidae	Megatominae	*Anthrenus museorum*	NC_063826	Circular	15,555	unpublished
Dermestidae	Megatominae	*Trogoderma granarium*	NC_053875	Circular	15,505	[37]
Dermestidae	Megatominae	*Trogoderma variabile*	MG011537	Linear	15,592	[41]
Dermestidae		*Dermestidae* sp. *GENSP01*	JX412839	Linear	12,521	unpublished
Dermestidae	Attageninae	*Attagenus unicolor japonicus*	OP235946	Circular	15,462	[37]
Dermestidae	Attageninae	*Attagenus augustatus augustatus*	MT113339	Circular	15,538	[37]
Dermestidae	Attageninae	*Attagenus hottentotus*	JX412837	Linear	14,309	unpublished
Derodontidae		*Derodontidae* sp. *BMNH 899913*	KX035159	Circular	16,711	unpublished
Nosodendridae		*Nosodendron* sp. *BMNH 840466*	KX035152	Circular	16,704	unpublished
Nosodendridae		*Nosodendron fasciculare*	KX087322	Linear	15,896	unpublished
Ptinidae	Xyletininae	*Lasioderma* sp. *EH001xFAY0068*	OQ716351	Linear	15,344	unpublished
Ptinidae	Xyletininae	*Lasioderma serricorne 1*	NC_038197	Circular	14,476	[42]
Ptinidae	Xyletininae	*Lasioderma serricorne 2*	MF417629	Circular	15,958	[43]
Ptinidae	Xyletininae	*Lasioderma serricorne 3*	MT254408	Circular	15,009	unpublished
Ptinidae	Xyletininae	*Lasioderma serricorne 4*	MH817138	Circular	14,550	[35]
Ptinidae	Xyletininae	*Lasioderma serricorne 5*	MG592705	Circular	14,476	[42]
Ptinidae	Xyletininae	*Lasioderma redtenbacheri*	KX087303	Linear	15,042	unpublished
Ptinidae	Ptininae	*Gibbium aequinoctiale*	KY549398	Circular	17,020	this study
Ptinidae	Anobiinae	*Anobiinae sp. BMNH 1274383*	KT696225	Circular	18,471	unpublished
Ptinidae	Anobiinae	*Gastrallus laevigatus*	KX087292	Linear	15,361	unpublished
Ptinidae	Anobiinae	*Anobiinae* sp. *GENSP01*	JX412730	Linear	12,786	unpublished
Ptinidae	Anobiinae	*Stegobium paniceum 1*	NC_036678	Circular	15,271	[44]
Ptinidae	Anobiinae	*Stegobium paniceum 2*	MK947052	Circular	15,474	unpublished
Ptinidae	Anobiinae	*Stegobium paniceum 3*	MH817140	Linear	14,101	[35]
Ptinidae	Anobiinae	*Stegobium paniceum 4*	KX819317	Circular	15,271	[44]
Outgroups		*Rhyzodiastes puetzi*	NC_067745	Circular	16,793	unpublished
		*Dineutus mellyi*	NC_054236	Circular	16,123	[45]

Note: “Circular” indicates the mitogenome was complete; “Linear” indicates the mitogenome was incomplete.

## 3. Results

### 3.1. Mitogenome Description

**Figure 1:**
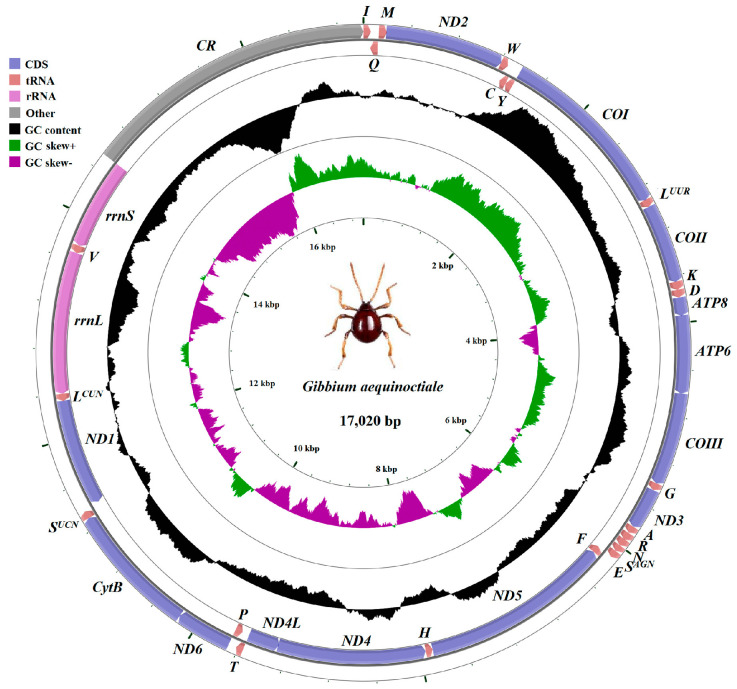
Mitogenome of *G. aequinoctiale*. The circular diagram was drawn using CGView. Arrows are utilized to denote the directionality of gene transcription. Gene name abbreviations are as follows: *ATP6* and *ATP8* represent ATP synthase subunits 6 and 8, *CytB* represents cytochrome b, *COI*–*III* represent cytochrome oxidase subunits 1–3, *ND1*–*6* and *ND4L* represent NADH dehydrogenase subunits 1–6 and 4L, and *rrnL* and *rrnS* represent large and small rRNA subunits. tRNA genes are designated by their corresponding one-letter amino acids. CR denotes the control region. The GC content was illustrated by utilizing a black-sliding window, focusing on its deviation from the overall average GC content. Moreover, GC-skew was portrayed by representing its divergence from the mean GC-skew of the entire sequence.

The mitogenome of *G. aequinoctiale* is 17,020 bp in length. This mitogenome contains a set of 37 representative metazoan mitochondrial genes (comprising 13 PCGs, 22 tRNAs, and two rRNAs) and a major non-coding region (CR, 2490 bp), which is situated in the interspace of small rRNA subunits (*rrnS*) and *trnI* (Figure 1 and Table 2). The mitogenome arrangement is shown in Figure 1 and appears identical to most other coleopteran mitogenomes. In contrast to the minority strand, which encodes 14 genes, the majority strand encodes 23 genes.

**Figure 2:**
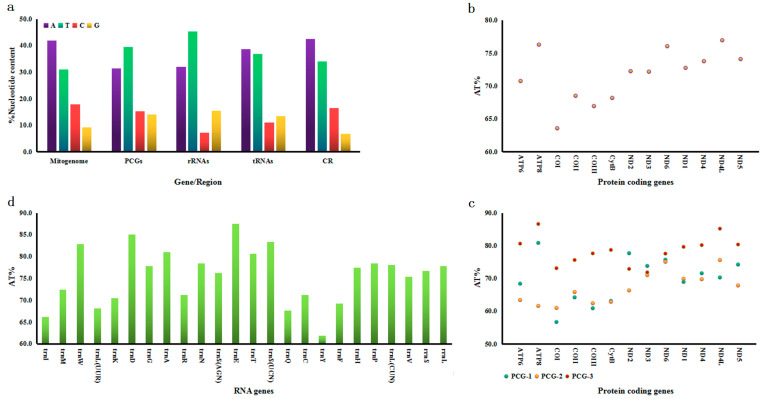
Nucleotide compositions for every part of the *G. aequinoctiale* mitogenome. (**a**) Nucleotide content (%) for genes or regions; (**b**) AT content for each PCG; (**c**) AT contents of three codon positions for each PCG; (**d**) AT contents for each tRNA and each rRNA.

The overall adenine–thymine (A + T) content across the entire mitogenome is 72.8% (A = 41.9%; T = 30.9%; C = 18.0%; G = 9.2%) on the majority strand (Figure 2a). The nucleotide composition displayed different strand bias patterns in the *G. aequinoctiale* mitogenome. PCGs and rRNAs demonstrated moderate T-skew, while tRNA genes and the CR exhibited A-skew. By contrast, PCGs displayed a marginal C-skew, the CR exhibited a prominent C-skew, and rRNAs and tRNAs demonstrated distinct G-skew (Figure 2a).

### 3.2. Protein-Coding Genes (PCGs)

**Figure 3:**
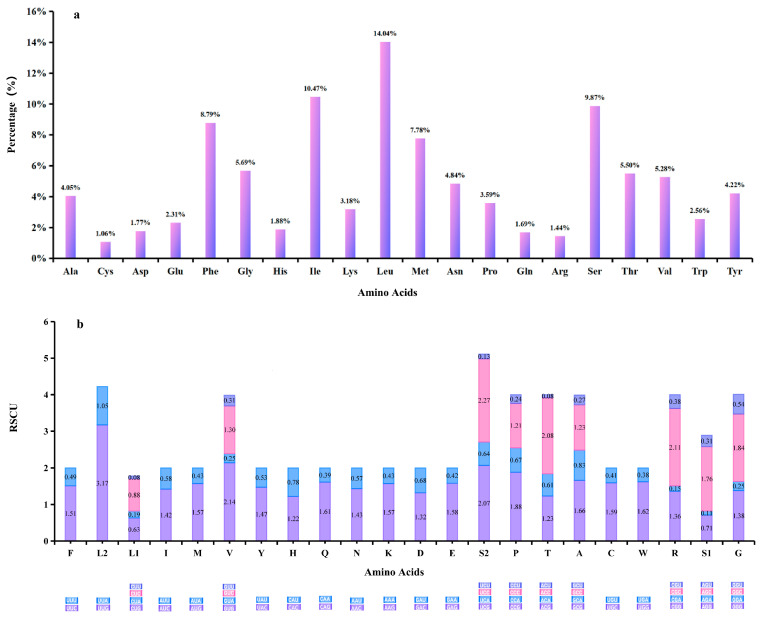
Amino acid composition and RSCUs of all PCGs in the *G. aequinoctiale* mitogenome. (**a**) Amino acid composition; (**b**) RSCUs.

The total A + T proportion across all PCGs reached 70.8%, with *ND4L* exhibiting the highest proportion at 77.0% and *COI* the lowest at 63.5% (Figure 2b). Notably, the third codon position exhibited the highest A + T content among all PCGs, except for *ND2* and *ND3* (Figure 2c). Twelve PCGs initiated with the canonical ATN codons: five started with ATG, four with ATT, two with ATA, and one with ATC, all of which are responsible for encoding either Met (M) or Ile (I). Furthermore, 10 PCGs possessed a full stop codon, with nine being TAA and one being TAG (Table 2). The predominant amino acids in the *G. aequinoctiale* mitochondrial PCGs are Leu (14.0%), Ile (10.5%), Ser (9.9%), and Phe (8.8%), accounting for 43.2% (Figure 3a). The genome-wide A + T bias is mirrored in the codon usage patterns of the *G. aequinoctiale* mitogenome. Analyses of RSCUs have revealed that codons featuring an A or T at the third position are consistently overrepresented compared to other synonymous codons (Figure 3b). Additionally, it has been noted that among all codons, TTT (coding for Phe), TTA (coding for Leu), ATT (coding for Ile), and ATA (coding for Met) exhibit the highest usage frequencies within the *G. aequinoctiale* PCGs (Figure 3b). Notably, all of these codons are composed solely of A or T nucleotides, indicating a biased preference for A and T nucleotides in the codon usage.

### 3.3. tRNA and rRNA Genes

**Figure 4:**
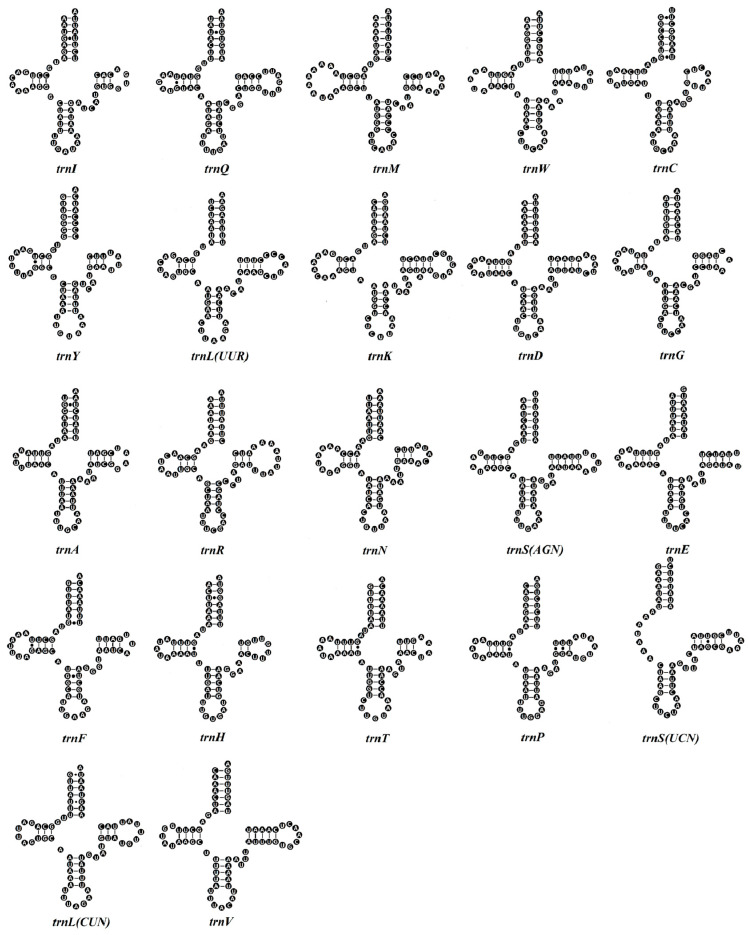
Inferred cloverleaf structures for 22 tRNAs in the *G. aequinoctiale* mitogenome. Lines represent inferred Watson–Crick bonds, whereas dots represent GU bonds.

Within the *G. aequinoctiale* mitogenome, 22 tRNA genes are distributed between the rRNA and PCGs. Specifically, there are two distinct tRNA genes coding for Ser and Leu, whereas each of the remaining amino acids is encoded by a singular tRNA gene locus. All tRNA gene loci and their secondary structural architectures were computationally delineated using tRNAscan-SE v1.21, with the exception of *trnR*, *trnH*, and *trnS^(AGN)^* in *G. aequinoctiale*, whose identification was achieved by conducting sequence comparison with previously published coleopteran mitogenomes (Table 2 and Figure 4). The tRNAs of *G. aequinoctiale* exhibit a size variation spanning from 59 bp for *trnS^(AGN)^* to 71 bp for *trnK*. The overall A + T bias in tRNA genes is 70.8%, with *trnE* showing the highest A + T enrichment (87.5%) and *trnY* exhibiting the lowest A + T content (61.9%). The A + T composition is 70.8% for total tRNA genes, with *trnE* having the highest (87.5%) and *trnY* the lowest (61.9%) (Figure 2a,d). Among 22 tRNA genes, six tRNA genes showed higher levels of A + T content (AT% > 80.0%), and only one of them was located on the minority strand (Figure 2d and Table 2).

The 22 tRNAs of *G*. *aequinoctiale* display conserved stem–loop architecture, with a uniform 7 bp aminoacyl acceptor stem and a 5 bp anticodon stem paired with a 7 nt anticodon loop, while non-canonical regions (D-arm, TΨC-arm, variable loop) exhibit length polymorphism and nucleotide substitution hotspots. All tRNAs of *G. aequinoctiale* are capable of adopting the characteristic clover-leaf structure, with the exception of *trnS^(AGN)^*, whose dihydrouridine (DHU) arm adopts a single-stranded loop topology (Figure 4). The same irregularity is observed in the *trnS^(AGN)^* of other insect mitogenomes [46]. Twenty-two tRNAs of *G. aequinoctiale* harbor a total of nine non-canonical base pairs in the stem region: five U-U, two A-A, and two A-C (Figure 4).

Consistent with the conserved genomic architecture of insect mitogenomes, both *rrnL* and *rrnS* were identified in *G. aequinoctiale*. The genomic loci of *rrnS* and *rrnL* were mapped to the intergenic regions bounded by *trnL^(CUN)^*-to-*trnV* and *trnV*-to-CR junctions, respectively (Figure 1 and Table 2). In *G. aequinoctiale*, the *rrnL* exhibits a length of 1270 bp, accompanied by an A + T content of 77.9%. The *rrnS*, on the other hand, has a length of 793 bp and is also biased to A and T in nucleotide composition (76.7%) (Figure 2d and Table 2).

### 3.4. Control Region (CR)

**Figure 5:**
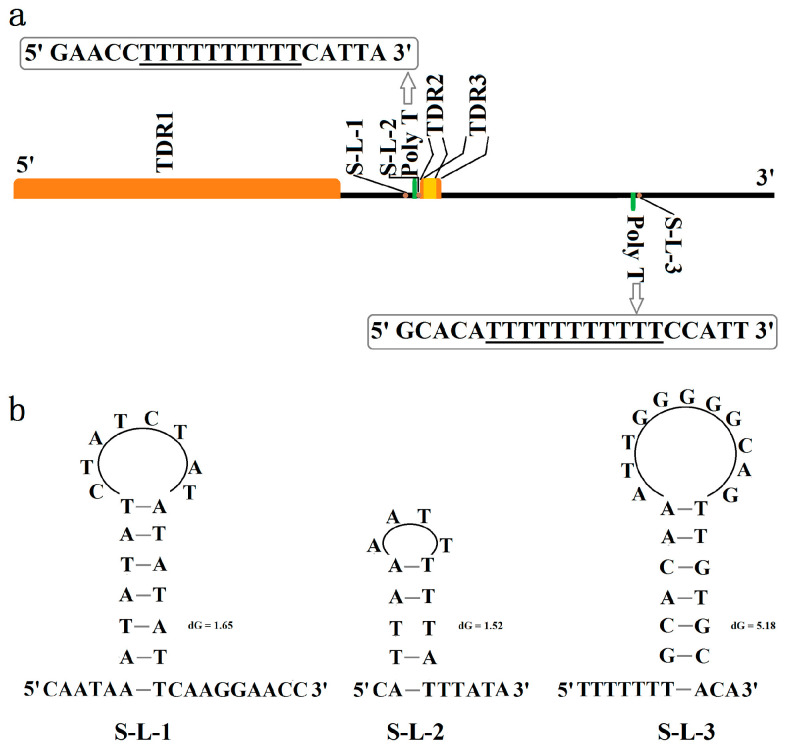
Structural feature of the control region in the *G. aequinoctiale* mitogenome. (**a**) Basic structure (TDR: tandem repeat, Poly-T: polythymine stretch, S–L: stem–loop); (**b**) putative stem–loop structure in the flanking region of polythymine stretch.

In *G. aequinoctiale*, the CR is positioned between *rrnS* and *trnI*. This entire region spans 2490 bp and exhibits a relatively high A + T nucleotide composition of 76.6% (Figure 2a), with three identifiable repeat regions, as frequently found in other coleopteran mitogenomes (Figure 5a). The region harbors a substantial tandem repeat that consists of approximately 2.3 nearly identical copies, each spanning 473 base pairs (bp) (with a similarity of 96%). The total length of this tandem repeat is 1074 bp, and it commences from nucleotide position 14,531 bp within the *G. aequinoctiale* mitogenome. The repetitive motif exhibits a nucleotide composition of 75.8% A + T (Table 3). Moreover, the control region contains another two tandem repeat (TDR) regions that share an overlapping region (from 15,880 to 15,918), and both tandem repeat units have strong bias to A and T in nucleotide composition (100% for TDR2 and 95.7% for TDR3), whereas the identities between each copy are slightly low in two tandem repeats (89% for TDR2 and 80% for TDR3) (Table 3). Additionally, all three motif sequences have the capacity to be folded into secondary structures featuring one or more stem–loops (Figure S1).

Excluding the aforementioned repeats, the predominant portion of the *G. aequinoctiale* CR consists of unique sequences. Nevertheless, it incorporates a 10 bp and 11 bp Poly-T on the majority strand and the minority strand, respectively, along with several microsatellite-like TA sequences dispersed across the entire region. The Poly-T on the minority strand starts at bp 16,558 and near the *trnI* gene. Moreover, the 5′ flanking region of Poly-T in both strands and the 3′ flanking region of Poly-T in the majority strand can form the stem–loop structure (Figure 5b).

### 3.5. Phylogenetic Analyses

**Figure 6:**
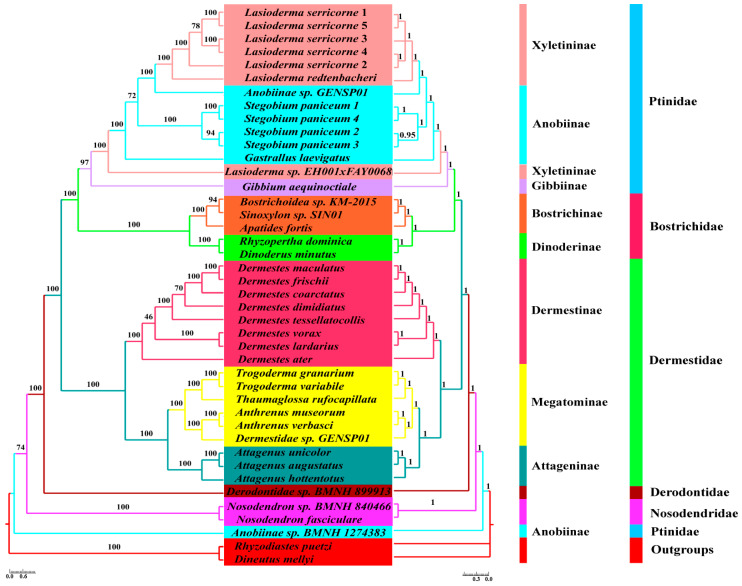
Phylogenetic tree for Bostrichiformia based on mtDNA using the BI and ML methods. Node numbers show posterior probabilities (**right**) and bootstrap support values (**left**). The scale bar represents the number of substitutions per site.

The mitogenome has been extensively employed to elucidate the phylogenetic relationships within diverse taxonomic groups of Coleoptera [8,9,10,11,12,13,14,15,16,17,18]. However, the inner phylogenetic relationship for the infraorder Bostrichiformia has received limited attention based on either morphological evidence or molecular evidence. To confirm the monophyletic status of each family and their phylogenetic positions within Bostrichiformia, we used four mitochondrial datasets (PCG, mtDNA, PCG12, and mtDNA12) from 40 species representing five families to reconstruct the phylogenetic tree using BI and ML methods (Figure 6 and Figure S2).

Our analyses, which were conducted based on four datasets, revealed well-supported groups and evolutionary associations across dominant lineages within the infraorder Bostrichiformia (Figure 6 and Figure S2). In this research, the monophyly of the four families Nosodendridae, Derodontidae, Dermestidae, and Bostrichidae was supported by all phylogenetic analyses (Figure 6 and Figure S2). However, the monophyly of Ptinidae was not supported, because *Anobiinae* sp. BMNH 1274383 was not clustered into the major branch of Ptinidae and formed an independent clade, and it was conventionally demonstrated to be positioned at the base of the Bostrichiformia (Figure 6).

## 4. Discussion

### 4.1. Mitogenome Characteristics

The mitogenome of *G. aequinoctiale* spans 17,020 bp, falling within the length range observed for the other mitogenomes of Bostrichiformia that have been sequenced. A standard set comprising 37 genes and a single CR was identified within this mitogenome, and the gene order exhibited congruence with that of other related species [8]. The mitogenomic nucleotide of *G. aequinoctiale* revealed a significantly elevated A + T proportion, with a dominant bias toward A and T across the three categories of genes and the CR. In the majority of PCGs, the third codon position exhibits the highest percentage of AT nucleotides. This phenomenon is presumably attributed to the elevated demand of PCGs for codons that select adenine (A) or uracil (U) as their third codon position. The CR is characterized by a higher A + T proportion, implying a potential link between its increased mutation rate and the higher A + T content, potentially operating under diminished selective pressure [47]. The AT content trends observed in the majority of mitogenome partitions may be attributed to the influence of respiratory processes, the transcription-coupled repair mechanism, and deamination reactions [48,49].

All PCGs commenced with the canonical ATN-initiated translation initiation codons, with the exception of *COI*, where the identification of a definitive start codon proved challenging. Previous studies have documented instances of aberrant translational initiation sites for *COI* across various insect orders, encompassing Diptera, Lepidoptera, Orthoptera, and Coleoptera. The hypothesis has been proposed that the optimal strategy for *COI* start codon selection involves maximizing coding sequence density while preventing transcriptional conflicts [50]. They further proposed the codons AAT or AAC encoding Asn as the start codon for *COI*, exhibiting a high degree of conservation across various divergent superfamilies within the suborder Polyphaga [50]. In *G. aequinoctiale*, we conducted an alignment with amino acid sequences translated for *COI* from other polyphagan species. Through this analysis, we deduced that the putative start codon for *COI* is AAT. TAA emerged as the predominant stop codon, whereas the TAG termination codon was observed only once. Additionally, an incomplete termination codon T in three PCGs (*COII*, *ND3,* and *ND5*) was detected. Post-transcriptional poly(A) tail addition triggers a templated elongation of the 3′ UTR, resulting in the de novo synthesis of the TAA termination triplet via runoff transcription or template-dependent nucleotide incorporation [51].

### 4.2. Non-Coding Region Analyses

Similar to the majority of insect mitogenomes, the mitogenome of *G. aequinoctiale* exhibits a compact structure and contains a cumulative length of 31 base pairs (bp) of intergenic spacer sequences. These sequences are dispersed across seven distinct regions, with sizes varying from 1 bp to 17 bp. The maximal intergenic region is intercalated between *trnS^(UCN)^* and *ND1* (Table 2). An intergenic spacer of comparable size, positioned between *trnS^(UCN)^* and *ND1*, exhibits widespread conservation in most sequenced coleopteran insects [9]. In a subtle deviation from the commonly observed 5 bp motif sequence (TAGTA) within the intergenic spacer of most coleopteran mitogenomes, a motif sequence (TACTA) has been observed in *G. aequinoctiale*, and this 5 bp motif is hypothesized to act as a cis-regulatory element mediating mtTERM-dependent transcription termination [52].

All TDRs within the CR exhibited an exceptionally high degree of sequence homology among repeat units, exceeding 95.0%. This observation may suggest that the copy turnover rate exceeds the nucleotide substitution rate, because the evolutionary trajectory of repetitive elements is primarily governed by the copy turnover rate relative to the nucleotide mutation rate [53]. Additionally, the stem–loop structures generated by the motif sequence within TDRs as a result of strand slippage may promote the formation of tandem repeats [53]. One long Poly-T was detected on the majority and minority strands, respectively. The extended Poly-T sequence located in proximity to the trnI gene on the minority strand exhibits a relatively high degree of conservation across various insect species. It has been postulated that this Poly-T sequence may act as a cis-regulatory element by either modulating transcription factor binding or facilitating replication initiation [54]. Furthermore, these potential stem–loop structures adjacent to the Poly-T region are promising candidates that could participate in the initiation of mitogenome replication.

### 4.3. Phylogenetic Relationship for Bostrichiformia and Ptinidae

According to Zahradník and Háva (2014), Bostrichiformia comprises seven families, Bostrichidae, Dermestidae, Ptinidae, Endecatomidae, Derodontidae, Nosodendridae, and Jacobsoniidae, among which the latter four families are all very small families [20]. Among families within the Bostrichiformia, our trees provided a topology of (Nosodendridae + (Derodontidae + (Dermestidae + (Bostrichidae + Ptinidae)))) (Figure 6), which seems to be somewhat inconsistent with previous studies, in which the infraorder Bostrichiformia is divided into two superfamilies, Derodontoidea and Bostrichoidea, or two series, Derodontiformia and Bostrichiformia [19]. Three subfamilies within Ptinidae were included in this study, and none of the phylogenetic analyses recovered monophyly for Anobiinae; similarly, Xyletininae was not supported as a monophyletic group across all analyses (Figure 6 and Figure S2). Nevertheless, Ptininae formed an independent clade, but with only one representative species. This outcome is consistent with the phylogenetic topology derived from concatenated molecular markers comprising *COI*, *rrnL,* and *28srRNA* [55], in which most subfamilies within Ptinidae do not exhibit monophyly, such as Gibbiinae, Dorcatominae, and the three subfamilies in this study. This research demonstrated that Bostrichidae forms a monophyletic group and is validated as a sister lineage to the major branch of Ptinidae, which was seemingly consistent with numerous prior investigations [12,14,56]. Two subfamilies, Dinoderinae and Bostrichinae, within Bostrichidae formed independent clades in this study. All analyses undertaken in this study unequivocally upheld the monophyly of Dermestidae and a family relationship in it, ((Attageninae + Megatominae) + Dermestinae) (Figure 6 and Figure S2), in accordance with the phylogenetic inference for the family Dermestidae derived from the 13 concatenated PCGs datasets [40]. Consistent with the previous study, the Dermestidae clustered with this large clade consisted of Ptinidae and Bostrichidae as sister groups. Derodontidae, which comprised one representative species, formed an independent clade. Two species from the family Nosodendridae also clustered into an independent branch as a monophyletic group. Both Derodontidae and Nosodendridae occupied the basal position within the phylogenetic framework of the infraorder Bostrichiformia and were not gathered as sister groups, regardless of the data matrices we assessed (Figure 6 and Figure S2).

In this study, we assessed and analyzed the influence of four datasets and two analytical methods on reconstructing the phylogeny of Bostrichiformia. The genes that constitute the mitogenome exhibit a relatively rapid evolutionary rate, and there are variations in this rate across different mitochondrial genes. Two rRNAs exhibit a relatively stable evolutionary rate, in contrast to the 13 PCGs, which experience a more accelerated evolutionary process. Accordingly, it can be speculated that site-specific rate heterogeneity across mitochondrial loci may influence phylogenetic reconstruction [14]. Overall, the phylogenetic inferences based on mtDNA (including two rRNAs) and PCG seemingly display similar family-level and subfamily-level relationships of Bostrichiformia. Nevertheless, compared to PCG, mtDNA showed slightly better balancing of heterogeneity in evolutionary rates among different mitochondrial PCGs; this was reflected in the bootstrap support of the inferred high-level phylogeny of Bostrichiformia. The bootstrap support supporting the major lineage for Ptinidae and the bootstrap support among Derodontidae and Nosodendridae and other families in Bostrichiformia are relatively higher. Furthermore, the analytical methods can also substantially influence phylogenetic inference. In contrast to the ML tree, the BI tree demonstrated superior nodal support and supported the monophyly of most families and subfamilies well. The node support values of BI trees consistently surpassed those of ML trees, particularly within branches with relatively low bootstrap support in the ML tree. This observation was in accordance with prior research outcomes. Phylogenetic analysis of Coleoptera based on mitogenomic data is frequently significantly impacted by heterogeneity in evolutionary rates [56]. The issue of compositional heterogeneity has long been recognized as a significant contributor to long-branch attraction, and it is commonly believed that eliminating third codon positions can mitigate this problem. This study explored the influence of the fast-evolving third site in PCGs in clarifying the phylogeny of Bostrichiformia, suggesting PCGs including the third codon positions demonstrate a stronger capability to resolve the majority of the anticipated main clades compared to PCG12 at the family and subfamily levels. This phenomenon is primarily attributable to the increased quantity of polymorphic loci, whereas each locus exhibits a higher information density on average.

## 5. Conclusions

This study presents the entire mitogenome of *G. aequinoctiale*, and this is the first reference mitogenome for the subfamily Ptininae. The 17,020 bp long *G. aequinoctiale* mitogenome exhibits a gene repertoire characteristic of metazoan mitogenome signatures and a gene ordering that is the same as most other insect mitogenomes. AAT was inferred as the potential initiation codon for *COI*. Three repeat regions, two poly-T, and stem–loop structures at the flanking regions of Poly-T were observed in the 2490 bp long control region (CR). The nucleotide content showed a heavy bias toward A and T for every part of the total mitogenome. Furthermore, phylogenetic relationships among Bostrichiformia were investigated, and all families of Bostrichiformia involved in this study were verified as monophyly except for Ptinidae. Bostrichidae, Ptinidae, and Dermestidae clustered as the major clade in Bostrichiformia, among which our analyses consistently indicated that Dermestidae occupied the basal position and the sister lineage with Bostrichidae and Ptinidae. However, the monophyletic status of Ptinidae and the phylogenetic relationships among Ptinidae at various hierarchical levels need to be further studied by increasingly dense taxon sampling.

## Figures and Tables

**Table 2:** Annotation of the G. aequinoctiale mitogenome.

Gene (Region)	Coding Strand	Position	Intergenic Gaps	Start Codon	Stop Codon	Anticodon	Length (bp)	AT%
*trnI*	+	1–62				GAT	62	66.1
*trnQ*	−	60–127	3			TTG	68	67.6
*trnM*	+	131–199				CAT	69	72.5
*ND2*	+	194–1204		ATT	TAA		1011	72.2
*trnW*	+	1203–1266				TCA	64	82.8
*trnC*	−	1259–1317				GCA	59	71.2
*trnY*	−	1318–1380	1			GTA	63	61.9
*COI*	+	1382–2917		AAT	TAA		1536	63.5
*trnL^UUR^*	+	2913–2978				TAA	66	68.2
*COII*	+	2978–3653		ATG	T		676	68.5
*trnK*	+	3651–3721				CTT	71	70.4
*trnD*	+	3721–3787				GTC	67	85.1
*ATP8*	+	3788–3943		ATT	TAA		156	76.3
*ATP6*	+	3940–4602		ATA	TAA		663	70.7
*COIII*	+	4602–5390		ATG	TAA		789	66.9
*trnG*	+	5390–5452				TCC	63	77.8
*ND3*	+	5453–5804		ATC	T		352	72.2
*trnA*	+	5805–5867	3			TGC	63	81.0
*trnR*	+	5871–5936				TCG	66	71.2
*trnN*	+	5934–5998				GTT	65	78.5
*trnS^AGN^*	+	5999–6057				AGT	59	76.3
*trnE*	+	6058–6121				TTC	64	87.5
*trnF*	−	6120–6184				GAA	65	69.2
*ND5*	−	6185–7889		ATT	T		1705	74.1
*trnH*	−	7890–7951	3			GTG	62	77.4
*ND4*	−	7955–9280		ATG	TAA		1326	73.8
*ND4L*	−	9274–9555	2	ATG	TAA		282	77.0
*trnT*	+	9558–9619				TGT	62	80.6
*trnP*	−	9620–9684	2			TGG	65	78.5
*ND6*	+	9687–10,166		ATT	TAA		480	76.0
*CytB*	+	10,166–11,302		ATG	TAA		1137	68.2
*trnS^UCN^*	+	11,301–11,366	17			TGA	66	83.3
*ND1*	−	11,384–12,337		ATA	TAG		954	72.7
*trnL^CUN^*	−	12,335–12,398				TAG	64	78.1
*rrnL*	−	12,399–13,668					1270	77.9
*trnV*	−	13,669–13,737				TAC	69	75.4
*rrnS*	−	13,738–14,530					793	76.7
Control region	+	14,531–17,020					2490	76.6

Note: The “+” sign denotes the majority DNA coding strand. The “−” sign denotes the minority DNA strand.

**Table 3:** Statistics of the TDR sequence in the G. aequinoctiale control region.

ID of TDR	Repeat Region in CR	Period Size	Copy Number	Percent Matches	AT%
TDR1	1–1074	473	2.3	96	75.8
TDR2	1339–1388	23	2.1	89	100
TDR3	1350–1405	21	2.5	80	95.7

Notes: ID, identification; TDRs, tandem repeats; CR, control region.

## Data Availability

The mitogenome sequencing data of *G. aequinoctiale* (KY549398) that support the findings of this study are openly available in the GenBank of NCBI at https://www.ncbi.nlm.nih.gov (accessed on 29 December 2024).

## Supplementary Materials

The following supporting information can be downloaded at: https://www.mdpi.com/article/10.3390/genes16050509/s1. Table S1: Primers used in this study; Figure S1: The potential secondary structure shaped by the motif sequence of TDRs; Figure S2: Phylogenetic tree of Bostrichiformia deduced from PCG, mtDNA12, and PCG12 using the BI and ML methods. Node designations display posterior probabilities alongside bootstrap support values. The scale bar denotes the rate of nucleotide substitutions per site. Data: The supplementary files include the alignment files and phylogenetic trees in this study.

## Abbreviations

The following abbreviations are utilized in this manuscript:

Mitogenome	mitochondrial genome
PCGs	protein-coding genes
tRNAs	transfer RNA genes
rRNAs	ribosomal RNA genes
CR	control region
TDRs	tandem repeats
PCR	polymerase chain reaction
*rrnS*	small rRNA subunits
*rrnL*	large rRNA subunits
PCG	13 PCGs
mtDNA	13 PCGs and two rRNAs
PCG12	13 PCGs excluding the 3rd codon position
mtDNA12	PCG12 and two rRNAs
AIC	Akaike information criterion
BIC	Bayesian information criterion
BI	Bayesian inference
ML	maximum likelihood
*ATP6* and *ATP8*	ATP synthase subunits 6 and 8
*COI*–*III*	cytochrome oxidase subunits 1–3
*CytB*	cytochrome b
*ND1*–*6* and *ND4L*	NADH dehydrogenase subunits 1–6 and 4L
RSCUs	relative synonymous codon usages
DHU arm	dihydrouridine arm
Poly-T	polythymine stretch
S–L	stem–loop
ID	identification
