# Supplementary figures and images for: Phylogenetic Analyses of Bostrichiformia and Characterization of the Mitogenome of Gibbium aequinoctiale (Bostrichiformia Ptinidae)
Source: Genes (Basel). 2025 Apr 28;16(5):509. doi: 10.3390/genes16050509 (PMC12111783; doi:10.3390/genes16050509)

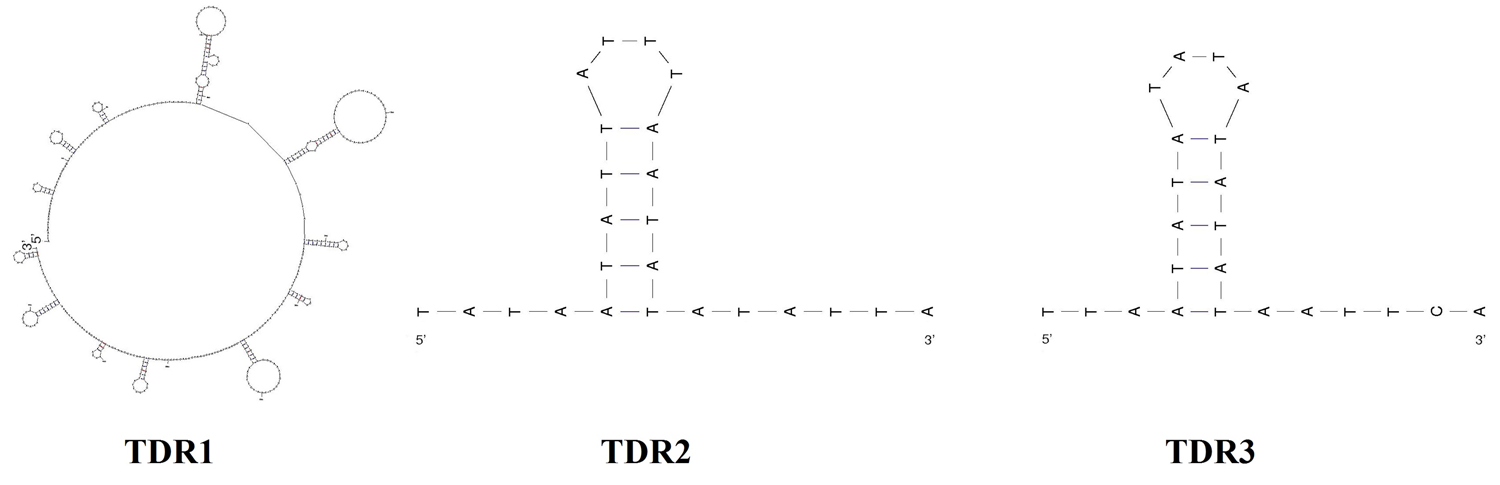

Supplement: Supplementary file 1 [file genes-16-00509-s001.zip › Figure S1.tif]

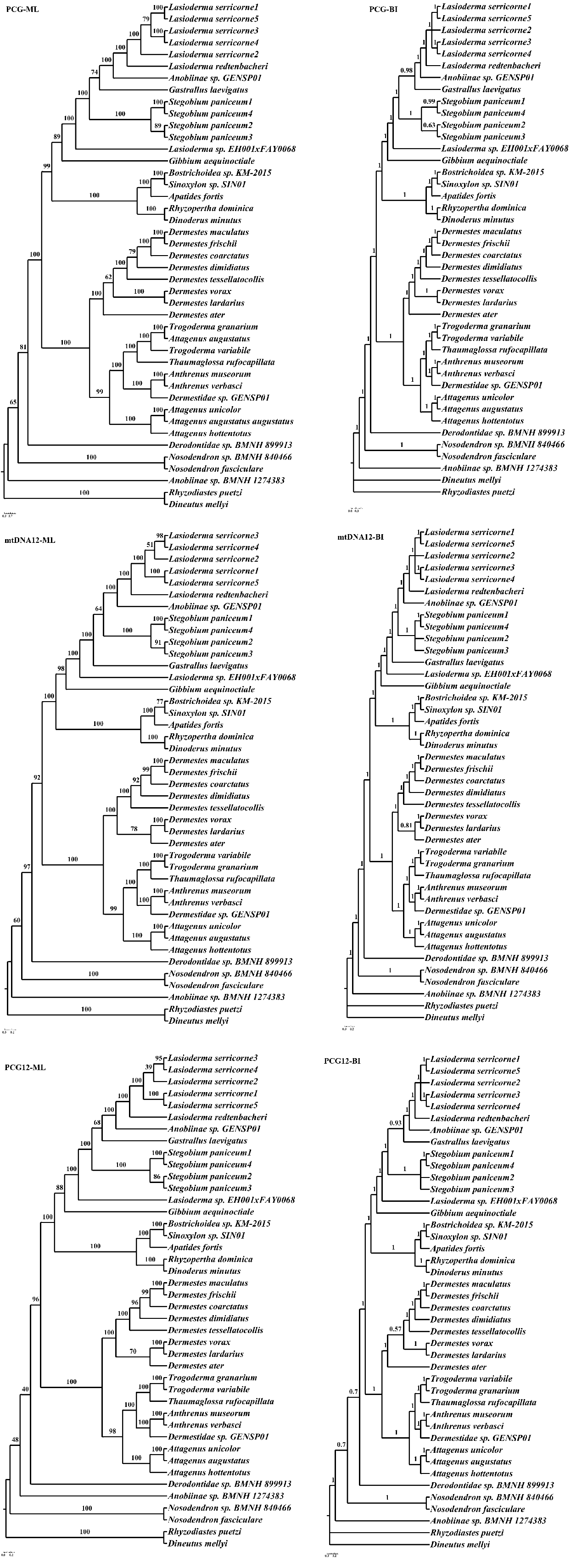

Supplement: Supplementary file 1 [file genes-16-00509-s001.zip › Figure S2.tif]
